# Four-Year Outcomes of Small Incision Lenticule Extraction for Extreme High Myopia and Myopic Astigmatism

**DOI:** 10.3389/fmed.2020.575779

**Published:** 2020-11-16

**Authors:** Fei Xia, Bing Qin, Jianmin Shang, Zhuoyi Chen, Xueyi Zhou, Jing Zhao, Xiaoying Wang, Xingtao Zhou

**Affiliations:** ^1^Eye Institute and Department of Ophthalmology, Eye & ENT Hospital, Fudan University, Shanghai, China; ^2^NHC Key Laboratory of Myopia, Fudan University, Shanghai, China; ^3^Key Laboratory of Myopia, Chinese Academy of Medical Sciences, Shanghai, China; ^4^Shanghai Research Center of Ophthalmology and Optometry, Shanghai, China

**Keywords:** small incision lenticule extraction, high myopia, visual outcome, corneal stability, wavefront aberrations

## Abstract

**Purpose:** To evaluate the long-term safety, efficacy, predictability, and stability of small incision lenticule extraction (SMILE) for the treatment of high myopia and myopic astigmatism >−10.0 D.

**Methods:** This was a prospective study that incorporated 35 consecutive patients (35 eyes) undergoing SMILE from September 2015 to March 2016. These patients had a mean preoperative spherical equivalent refraction of −10.06 ± 0.64 D. Patients were followed over a 4-year period and assessed for outcomes including uncorrected distance visual acuity (UDVA), corrected distance visual acuity (CDVA), manifest refraction, and corneal topography.

**Results:** At 4 years post-SMILE, respective efficacy and safety indices were 1.01 ± 0.19 and 1.07 ± 0.15. In total, 97% of operated eyes achieved an UDVA of 20/25 or better. ≥1 line was gained for 9 eyes (26%), with 25 eyes (71%) remaining stable. Twenty-four (69%) and 33 (94%) eyes, respectively, were within ±0.50 D and ±1.0 D of target refraction. From 3 months to 4 years postoperatively, a mean refractive regression of −0.22 D (−0.06 D per year) was detected, whereas no significant changes in mean corneal back curvature or posterior central elevation were detected (*P* = 0.617 and 0.754, respectively). We detected significant increases in higher-order aberrations (HOAs) of the anterior and total cornea (all *P* < 0.001), with spherical aberrations and vertical coma being particularly common, whereas posterior corneal HOA remained fairly stable (all *P* < 0.05).

**Conclusion:** SMILE is a safe, effective, predictable, and stable means of correcting high myopia and myopic astigmatism over a 4-year postoperative period.

## Introduction

The minimally invasive, flapless small incision lenticule extraction (SMILE) procedure offers a means of retaining superior corneal integrity relative to laser *in situ* keratomileuses (LASIK) while eliminating flap-related adverse effects. Since it was initially described in 2011 (1, 2), SMILE has been confirmed to be safe, efficacious, predictable, and stable in a range of studies (3–6). Following 2016 updates to the VisuMax femtosecond laser system software (Carl Zeiss Meditec AG, Jena, Germany), surgical indications for this approach were expanded to the treatment of myopia and myopic astigmatism of >-10.0 D, with this approach having been shown to be safe and effective at 6 and 15 months postoperatively by Qin et al. (7) and Yang et al. (8), respectively. However, it is essential that long-term observation of patients with high myopia treated via SMILE be conducted in order to evaluate the incidence of postoperative regression and ectasia. As such, the present study was designed to assess postoperative visual and refractive outcomes of SMILE-based correction of myopia and myopic astigmatism of over −10 D, with the goal of evaluating the safety, efficacy, predictability, and stability of this procedure over a 4-year postoperative period.

## Materials and Methods

This was a prospective study that incorporated 35 consecutive patients (6 males, 29 females; 35 eyes) undergoing SMILE at the Eye and ENT Hospital of Fudan University (Shanghai, China) between September 2015 and March 2016. Patients had a mean age of 26.77 ± 5.54 years (range: 18 to 38) and a mean preoperative spherical equivalent (SE) refraction of −10.06 ± 0.64 D (range: −11.75 to −8.88 D) (Table 1).

**Table 1:** Baseline clinical and demographic characteristics.

**Parameters**	**Mean ± SD (Range)**
Gender (*n*/*n*)	6 males/29 females
Age (years)	26.77 ± 5.54 (18 to 38)
Sphere (D)	−9.53 ± 0.84 (−11.25 to −8.25)
Cylinder (D)	−1.09 ± 0.64 (−2.25 to 0)
SE (D)	−10.06 ± 0.74 (−11.75 to −8.88)
Axial length(mm)	27.15 ± 0.81 (25.51 to 29.67)
IOP (mmHg)	16.63 ± 2.43 (12.1 to 22.0)
CCT (μm)	544.60 ± 22.16 (504 to 609)
Optical zone (mm)	6.19 ± 0.19 (6.0 to 6.5)
Ablation depth (μm)	150.41 ± 6.06 (130 to 158)
Residual bed thickness (μm)	278.56 ± 17.74 (259 to 318)

*D, diopters; SE, spherical equivalent; CCT, central corneal thickness IOP, intraocular pressure*.

The ethics committee of the Eye and ENT Hospital, Fudan University approved the present study, which was consistent with the Declaration of Helsinki. All patients provided written informed consent.

Patients included in the present study met the following criteria: age ≥ 18 years; spherical refraction error > −8.0 D; sum spherical and cylinder refraction error > −10.0 D; stable refraction over a 2-year preoperative period; corrected distance visual acuity (CDVA) ≥ 20/25; residual corneal stromal bed thickness ≥ 250 μm; no contact lens utilization within 2 weeks preoperatively.

Patients were excluded from this study if they met the following criteria: patients suffered or were suspected to suffer from keratectasia; patients had undergone prior ocular surgery or trauma; patients suffered from severe dry eyes, retinal detachment, or other ocular conditions (not including myopia or astigmatism); patients suffered from connective tissue disorders or other systemic diseases. We additionally excluded any eyes that had a calculated postoperative residual stromal bed < 250 μm.

### Measurements

Standard clinical preoperative refractive surgical procedures were conducted for all patients, including uncorrected distance visual acuity (UDVA), CDVA, objective and manifest refraction, intraocular pressure, axial length, slit-lamp, and Goldmann three-mirror contact lens assessments. Corneal topography and wavefront aberrations were assessed with a Pentacam Scheimpflug imaging instrument (Pentacam HR, Type 70900, Wetzlar, Germany).

Patients underwent follow-up analyses at 3 months, 6 months, 1 year, and 4 years postoperatively, at which time objective and manifest refraction, UDVA, CDVA, corneal topography, and wavefront aberrations were assessed as above.

Pentacam Scheimpflug imaging was employed to evaluate mean corneal back curvature (MCBC), central corneal thickness (CCT), mean corneal front curvature (MCFC), and posterior central elevation (PCE). MCFC, MCBC, and PCE were delineated in the central 4-mm area above the 8-mm reference best-fit sphere. ΔPCE was the difference between pre- and post-operative PCE based on the same reference best-fit sphere.

Wavefront aberrations of the anterior surface, posterior surface, and total cornea were assessed under standard scotopic light settings for a central 5-mm zone. Root mean square (RMS) values of corneal higher-order aberrations (HOAs) were analyzed, including HOAs up to fourth order, quatrefoil, and secondary astigmatism. Zernike polynomials were also used to evaluate coefficients of spherical aberration ($z_{4}^{0}$), the vertical coma ($z_{3}^{-1}$), the horizontal coma ($z_{3}^{1}$), the vertical trefoil ($z_{3}^{-3}$), horizontal trefoil ($z_{3}^{3}$).

### Surgical Procedures

A single experienced surgeon (XZ) conducted all SMILE procedures with a VisuMax femtosecond laser system (Carl Zeiss Meditec AG, Jena, Germany) as discussed previously (9). Operative parameters were as follows: pulse energy = 130 nJ, laser repetition rate = 500 kHz, lenticule diameter = 6.0–6.5 mm, cap thickness = 110–120 μm, cap diameter = 7.5 (larger than 1 mm of lenticule), side cut of 2 mm side cut (90°) at 12:00 clock.

Postoperatively, patients were administered topical 0.5% levofloxacin four times per day over a 7-day period and were administered artificial tears four times per day for 1 month or longer. In addition, patients were administered a 0.1% fluorometholone solution, initially dosing eight times per day and then tapering every 3 days down to once daily.

### Statistical Analysis

SPSS v. 23 (SPSS, Inc., IL, United States) was used for all statistical testing. Only the right eye from each patient was analyzed. Categorical variables are frequencies and percentages, while continuous variables are means ± standard deviation (SD). Data normality was evaluated via the Kolmogorov–Smirnov test, with paired Student's *t*-tests and Wilcoxon signed-rank tests being used to compare normally and non-normally distributed data between the 3-month and 4-year follow-up time points. Correlations between attempted and achieved SEs were assessed via Pearson correlation analyses. *P* < 0.05 was the significance threshold.

## Results

There was no incidence of intraoperative or postoperative complications in any of the 35 patients included in the present study. The axial length was 27.14 ± 0.82 mm preoperatively and 27.10 ± 0.90 mm 4 years postoperatively. There were no statistical difference comparing the preoperative axial length and 4 years postoperative one (*P* = 0.143). Four-year postoperative outcomes in these patients were analyzed as follows:

### Efficacy and Safety

**Figure 1:**
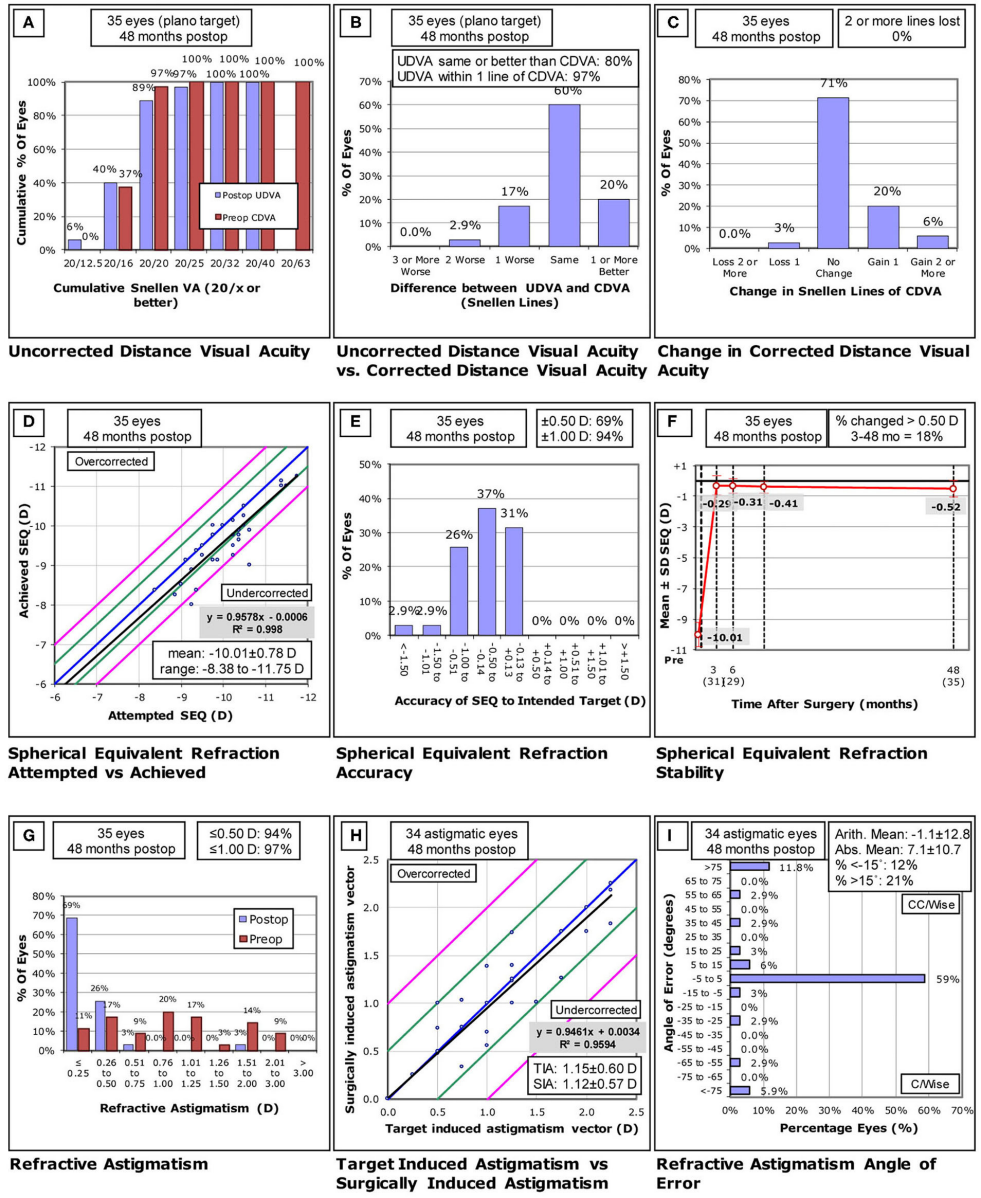
**(A-I)** Standard graphs of refractive surgery visual and refractive outcomes for 35 eyes at 4 years post-small incision lenticule extraction (SMILE). UDVA, uncorrected distance visual acuity; CDVA, corrected distance visual acuity; D, diopters; Postop, postoperative; Preop, preoperative; SEQ, spherical equivalent refraction; TIA, -target induced astigmatism; SIA, surgically induced astigmatism. (**(A)** Uncorrected distance visual acuity. **(B)** Uncorrected distance visual a-cuity vs corrected distance visual acuity. **(C)** Change in corrected distance visual acuity. **(D)** Spherical equivalent refraction attempted vs- achieved. **(E)** Spherical equivalent refractive accuracy. **(F)** Stability of spherical equivalent refraction. **(G)** Refractive astigmatism. **(H)** Target induced astigmatism vs surgically induced astigmatism. **(I)** Refractive astigmatism angle of error).

The efficacy and safety of the 35 eyes are shown in Figures 1A–C. Preoperative mean UDVA and CDVA were 1.36 ± 0.27 (range: 1.0 to 1.6) log MAR and −0.03 ± 0.06 (range: −0.1 to 0.1) log MAR, respectively. At the 4-year follow-up visit, postoperative mean UDVA was −0.03 ± 0.08 (range: −0.2 to 0.2) log MAR, and postoperative mean CDVA was −0.06 ± 0.07 (range: −0.2 to 0) log MAR. The efficacy and safety indices in these patients were 1.01 ± 0.19 and 1.07 ± 0.15, respectively.

### Predictability and Stability

Attempted and achieved SE correction values are shown in Figure 1D. In total, 24 (69%) and 33 (94%) eyes were within ±0.50 D and ±1.0 D of target refraction (Figure 1E). Between 3 months and 4 years postoperatively, mean SE changed from −0.29 ± 0.63 D to −0.52 ± 0.55 D, but these changes were not significant (*P* = 0.126), with a mean refractive regression of −0.22 D (−0.06 D/year) being observed over this same period (Figure 1F). Changes in SE refractive error were more than 0.5 D in 5 (16.1%) out of 31 eyes.

### Refractive Astigmatism

Refractive astigmatism results are shown in Figures 1G–I. In total, 94% (33 eyes) and 97% (34 eyes) of eyes exhibited postoperative astigmatism of ≤ 0.5 D and ≤ 1 D, respectively, and 59% (20 eyes) of eyes exhibited a refractive astigmatism angle of error < ±5°.

### Corneal Stability

Corneal stability analyses of Scheimpflug imaging are shown in Table 2. We observed significant increases in 4-year postoperative CCT and MCFC values relative to these same values at 3 months postoperatively (all *P* < 0.001), whereas MCBC, PCE, and ΔPCE were comparable at all preoperative and postoperative time points (all *P* > 0.05).

**Table 2:** Corneal stability analysis of Scheimpflug imaging.

**Parameters**	**Preoperative**	**3 Months**	**4 Years**	***P*** **value**		
				**3 Months vs Preop**	**4 Years vs Preop**	**4 Years vs. 3 Months**
CCT (μm)	545.31 ± 23.23	413.57 ± 20.62	426.22 ± 21.51	<0.001^*^	<0.001^*^	<0.001^*^
MCFC (D)	43.49 ± 1.32	36.37 ± 1.50	37.11 ± 1.40	<0.001^*^	<0.001^*^	<0.001^*^
MCBC (D)	−6.303 ± 0.252	−6.288 ± 0.236	−6.312 ± 0.217	0.48	0.617	0.206
PCE (μm)	1.094 ± 2.966	1.783 ± 3.605	1.281 ± 4.12	0.025	0.754	0.91
ΔPCE (μm)	/	−0.632 ± 2.454	0.316 ± 2.907	/	/	0.219

*CCT, central corneal thickness; MCFC, mean corneal front curvature (from central 4-mm-diameter cornea); MCBC, mean corneal back curvature (from central 4-mm-diameter cornea); PCE, posterior central elevation; ΔPCE, subtracting preoperative PCE from postoperative PCE*.

*Data are expressed as mean ± SD*.

* *Analyzed by t-test for paired samples (P <0.05)*.

### Corneal Wavefront Aberrations

Changes in corneal wavefront aberrations after SMILE are shown in Table 3. Significant increases in the anterior surface and total corneal HOAs were observed at 3 months and 4 years postoperatively relative to preoperative values, whereas posterior corneal HOA values did not increase (*P* = 0.525). Spherical aberration, vertical coma, and horizontal coma of the anterior surface and total cornea rose significantly with the exception of secondary astigmatism (*P* = 0.692) at 3 months post-SMILE relative to preoperative values. In addition, at 4 years postoperatively, spherical aberration, vertical coma, and secondary astigmatism of the anterior surface and total cornea all rose significantly (all *P* < 0.05) relative to preoperative values. Furthermore, vertical coma of the anterior surface and total cornea rose at 4 years postoperatively relative to 3 months postoperatively (all *P* < 0.05), whereas horizontal coma decreased significantly over this same period (*P* < 0.05). All other aberrations remained relatively stable over this period.

**Table 3:** Preoperative vs. postoperative corneal HOAs after small incision lenticule extraction (SMILE).

**Parameters**	**3 Months vs. Preop**			**4 Years vs. Preop**			**4 Years vs. 3 Months**		
	***n***	**Mean difference (μm)**	***P*-value**	***n***	**Mean difference (μm)**	***P*-value**	***n***	**Mean difference (μm)**	***P*-value**
Anterior surface
RMS HOA	31	0.411 ± 0.262	<0.001^*^	34	0.423 ± 0.261	<0.001^*^	31	0.02 ± 0.170	0.544
Spherical aberration	31	0.078 ± 0.136	0.006^*^	34	0.059 ± 0.088	0.001^*^	31	−0.023 ± 0.105	0.269
Coma (vertical)	31	−0.328 ± 0.306	<0.001^*^	34	−0.431 ± 0.266	<0.001^*^	31	−0.124 ± 0.225	0.007^*^
Coma (horizontal)	31	0.126 ± 0.215	0.006^*^	34	−0.039 ± 0.211	0.311	31	−0.130 ± 0.161	<0.001^*^
Trefoil (vertical)	31	0.030 ± 0.069	0.037^*^	34	−0.016 ± 0.070	0.194	31	−0.051 ± 0.080	0.002^*^
Trefoil (horizontal)	31	0.018 ± 0.076	0.245	34	0.014 ± 0.071	0.281	31	−0.009 ± 0.079	0.565
RMS tetrafoil	31	0.009 ± 0.040	0.235	34	0.024 ± 0.047	0.006^*^	31	0.016 ± 0.052	0.116
RMS secondary astigmatism	31	0.089 ± 0.081	<0.001^*^	34	0.063 ± 0.064	<0.001^*^	31	−0.011 ± 0.082	0.441
Posterior surface
RMS HOA	31	0.007 ± 0.021	0.17	34	−0.002 ± 0.017	0.525	31	−0.007 ± 0.02	0.081
Spherical aberration	31	0.007 ± 0.007	<0.001^*^	34	0.004 ± 0.008	0.003^*^	31	0 ± 0.011	0.835
Coma (vertical)	31	0.017 ± 0.022	0.001^*^	34	0.024 ± 0.022	<0.001^*^	31	0.005 ± 0.018	0.135
Coma (horizontal)	31	−0.008 ± 0.013	0.005^*^	34	−0.002 ± 0.015	0.551	31	0.006 ± 0.012	0.012^*^
Trefoil (vertical)	31	−0.022 ± 0.036	0.003^*^	34	0.002 ± 0.038	0.793	31	0.019 ± 0.045	0.037^*^
Trefoil (horizontal)	31	0.005 ± 0.029	0.367	34	0.004 ± 0.028	0.487	31	0.006 ± 0.029	0.263
RMS tetrafoil	31	0.008 ± 0.018	0.041^*^	34	0.003 ± 0.016	0.35	31	−0.005 ± 0.025	0.331
RMS secondary astigmatism	31	0.001 ± 0.013	0.692	34	−0.004 ± 0.008	0.01^*^	31	−0.003 ± 0.012	0.183
Total cornea
RMS HOA	31	0.405 ± 0.233	<0.001^*^	34	0.417 ± 0.224	<0.001^*^	31	0.028 ± 0.164	0.38
Spherical aberration	31	0.083 ± 0.137	0.004^*^	34	0.053 ± 0.096	0.003^*^	31	−0.026 ± 0.105	0.197
Coma (vertical)	31	−0.317 ± 0.296	<0.001^*^	34	−0.431 ± 0.256	<0.001^*^	31	−0.132 ± 0.233	0.006^*^
Coma (horizontal)	31	0.088 ± 0.170	0.018^*^	34	0.004 ± 0.273	0.939	31	−0.118 ± 0.175	0.001^*^
Trefoil (vertical)	31	0.001 ± 0.093	0.97	34	−0.020 ± 0.079	0.154	31	−0.043 ± 0.070	0.004^*^
Trefoil (horizontal)	31	0.021 ± 0.078	0.194	34	0.014 ± 0.083	0.356	31	−0.024 ± 0.085	0.161
RMS tetrafoil	31	0 ± 0.042	0.913	34	0.012 ± 0.050	0.17	31	0.005 ± 0.054	0.622
RMS secondary astigmatism	31	0.097 ± 0.091	<0.001^*^	34	0.067 ± 0.063	<0.001^*^	31	−0.008 ± 0.069	0.565

*HOA, total higher-order aberration; RMS, root mean square*.

*Data are expressed as mean ± SD*.

* *Analyzed by t test for paired samples (P <0.05)*.

## Discussion

In patients with high myopia undergoing SMILE treatment, long-term follow-up is essential to evaluate the potential for refractive regression and corneal ectasia. This study evaluated the safety, efficacy, predictability, and stability of SMILE procedure in correcting very high myopia over a 4-year follow-up period.

Herein, we determined that over a 4-year postoperative period, SMILE was associated with excellent efficacy and safety when used to treat extreme high myopia. This efficacy was supported by the fact that 89% of patients exhibited an UDVA of 20/20 or better, in line with prior studies (7, 8). With respect to safety, there was no incidence of any intraoperative or postoperative complications, and a single eye (2.9%) lost one line of CDVA, comparable to prior findings over a 1-year follow-up period. In their studies, Niu et al. (10) and Xia et al. (11) observed no lost CDVA in any eyes at 1-year (mean SE −7.39 D) and 3-year (mean SE of −8.11 D) follow-up time points, whereas other studies have exhibited poorer outcomes (4, 12–15), with 9–16% of eyes losing one or more lines of CDVA, potentially due to the presence of interlayer scattering, dry eyes, or the use of different VisuMax laser parameters (200 kHz). Moreover, a recent retrospective study (16) reported the 3-year results of SMILE in 495 eyes with very high myopia (mean spherical refraction error of −12.84 ± 2.47 D combined with mean astigmatism of −1.17 ± 1.34 D) and revealed that no significant differences were found in the efficacy and safety comparing the results of postoperative 1 month and 3 years. These results were relatively similar to our findings.

Concerning predictability, Pedersen et al. (17) determined that 78% and 90% of eyes were within ±0.50 and ±1.00 D at 3 years post-SMILE (mean SE of −7.30 D). In order to prevent under-correction, Burazovitch et al. (18) employed an 8% correction factor and found that 87% (100%) of eyes were within ±0.50 D (±1.00 D) (mean SE of−7.59 D) of refraction error, while Moshirfar et al. (19) found that in their experience, 100% of eyes that had undergone SMILE were within ±0.50 D, relative to just 66% of Toric implantable Collamer lens (ICL)-treated eyes (mean SE > −10.0 D). This led the authors to conclude that SMILE is superior to Toric ICL, although it is important to note that the SMILE group in this study was relatively small. Yang et al. (20) found 90 and 100% of eyes were within 0.5 D after SMILE and FS-LASIK during the 6-month observation period. They concluded that the predictability of SMILE was inferior than FS-LASIK due to the unprecise correction of the spherical power. In contrast, we found that 69% (94%) of the eyes were within ±0.5 D (±1.0 D) of the attempted correction at 4 years postoperatively, which was slightly inferior than the abovementioned studies. This discrepancy may be attributed to differential corneal wound healing response, more curvature change of corneal tissue, epithelial hyperplasia, and smaller optical zone of the corrections of high myopia, compared to the corrections of mild-to-moderate myopia. New customized nomograms could be considered to further improve the predictability of SMILE for very high myopia correction.

With respect to stability, two of the main factors influencing refractive regression are corneal epithelial remodeling and the stromal healing response (21). Correction of high myopia is associated with greater thickening of the corneal epithelium than is the correction of low myopia (22). In prior long-term studies, relatively mild regression has been reported following SMILE. Blum et al. (4), for example, detected a −0.48 D myopic regression at 5 years post-SMILE surgery and found the primary cause to be axial length elongation in 6/7 examined eyes, with central corneal steepening being the cause in the remaining eye. When these seven eyes were excluded from their analyses, a regression of 0.28 D was instead observed. Li et al. (23) observed myopic regression of −0.02 D (mean SE of −6.37 D) between 6 months and 5 years postoperatively, with this reduced regression being attributable to the fact that they were focused on the correction of low myopia. Additionally, Agca et al. (24) observed progressive regression of −0.26 D, −0.33 D, and −0.43 D at 1, 3, and 5 years post-SMILE, respectively, with these differences only being significant between the 1- and 5-year postoperative time points (mean SE of −7.47 D). This led the authors to conclude that regression began occurring at early postoperative stages, but did not become significant until after 5 years. We observed a mean regression of −0.22 D (−0.06 D per year). These differences may be attributable to our relatively small sample size, the stable myopic progression in these patients, and the small designated optical zone.

With respect to corneal stability, we observed significant changes in MCFC from 3 months to 4 years post-SMILE, in line with prior findings (25, 26). We additionally observed no changes in the posterior corneal surface following SMILE. Wang et al. (27) have also reported no changes in the posterior corneal surface at 1 year after SMILE in patients with moderate and high myopia. Zhou et al. (13) similarly observed stable posterior corneal elevation 2 years after SMILE for myopia higher than −10.0 D. Our results extended these findings, confirming that posterior corneal stability was evident at 4 years post-smile in patients treated for extreme myopia.

With respect to HOAs, we observed significant increases in anterior and total corneal aberrations over the 4-year follow-up period, with pronounced increases in vertical coma and spherical aberration. In contrast, posterior corneal HOAs were relatively stable over this 4-year period as compared to preoperative values. These findings are in line with the results of Jin et al. (28) and Wu et al. (29). We hypothesize that high preoperative SE is related to the observed anterior and total corneal aberrations. Jin et al. (28) similarly found that patients with high myopia exhibited significantly increased rates of surgically induced aberrations relative to patients with mild and moderate myopia. We also found that vertical coma rose significantly over this 4-year period, whereas horizontal coma nearly recovered to preoperative levels over this same time period. These vertical coma changes may be attributable to wound healing responses associated with the single superior incision involved in the SMILE procedure, with eyelids being likely to play a role during the postoperative period.

There are multiple limitations to the present study. For one, our sample size was relatively small owing to the limited number of patients with extreme high myopia eligible for SMILE surgery. In addition, we did not compare SMILE to LASIK, ICL, or other surgeries, and as such future comparisons of these approaches will be necessary. In addition, further research is required to explore the relationships between optical quality, intraocular scattering, and HOAs.

## Conclusions

Our findings confirm the long-term safety, efficacy, predictability, and stability of SMILE surgery as a means of correcting extreme myopia and myopic astigmatism of over −10 D.

## Data Availability Statement

The data analyzed in this study is subject to the following licenses/restrictions: Data analyzed during the current study are available from the corresponding author on reasonable request. Requests to access these datasets should be directed to Xingtao Zhou, doctzhouxingtao@163.com.

## Ethics Statement

The studies involving human participants were reviewed and approved by the ethics committee of the Eye and ENT Hospital, Fudan University. The patients/participants provided their written informed consent to participate in this study.

## Author Contributions

FX, BQ, and XiZ: concept and design. FX, BQ, JS, ZC, XuZ, and JZ: data collection and analysis. FX and BQ: writing the article. FX, BQ, XW, and XiZ: critical revision of the article. All authors: final approval of the article.

## Conflict of Interest

The authors declare that the research was conducted in the absence of any commercial or financial relationships that could be construed as a potential conflict of interest.
